# Preparation of monolayers of [Mn^III^_6_Cr^III^]^3+ ^single-molecule magnets on HOPG, mica and silicon surfaces and characterization by means of non-contact AFM

**DOI:** 10.1186/1556-276X-6-486

**Published:** 2011-08-08

**Authors:** Aaron Gryzia, Hans Predatsch, Armin Brechling, Veronika Hoeke, Erich Krickemeyer, Christine Derks, Manfred Neumann, Thorsten Glaser, Ulrich Heinzmann

**Affiliations:** 1 Molecular and Surface Physics, Faculty of Physics, Bielefeld University, Universitaetsstrasse 25, 33615 Bielefeld, Germany; 2 Inorganic Chemistry I, Faculty of Chemistry, Bielefeld University, Universitaetsstrasse 25, 33615 Bielefeld, Germany; 3 Electron Spectroscopy, Faculty of Physics, Osnabrueck University, Barbarastrasse 7, 49069 Osnabrueck, Germany

## Abstract

We report on the characterization of various salts of [**Mn^III^_6_Cr^III^]^3+ ^**complexes prepared on substrates such as highly oriented pyrolytic graphite (HOPG), mica, SiO_2_, and Si_3_N_4_. [**Mn^III^_6_Cr^III^]^3+ ^**is a single-molecule magnet, i.e., a superparamagnetic molecule, with a blocking temperature around 2 K. The three positive charges of [**Mn^III^_6_Cr^III^]^3+ ^**were electrically neutralized by use of various anions such as tetraphenylborate (BPh_4_^-^), lactate (C_3_H_5_O_3_^-^), or perchlorate (ClO_4_^-^). The molecule was prepared on the substrates out of solution using the droplet technique. The main subject of investigation was how the anions and substrates influence the emerging surface topology during and after the preparation. Regarding HOPG and SiO_2_, flat island-like and hemispheric-shaped structures were created. We observed a strong correlation between the electronic properties of the substrate and the analyzed structures, especially in the case of mica where we observed a gradient in the analyzed structures across the surface.

## Introduction

**Figure 1:**
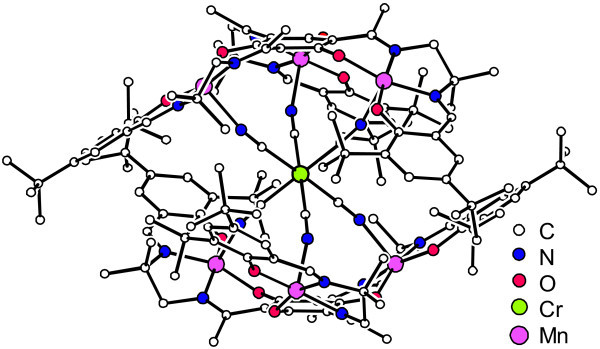
**Molecular structure of [Mn^III^_6_Cr^III^]^3+ ^in crystals of [Mn^III^_6_Cr^III^](BPh_4_)_3 _4MeCN 2Et_2_O **[6].

Current technology demands the development of smaller devices in various fields. The next step necessary involves reducing small bulk objects down the scale to where a single molecule has a specific task. Mn in this context is an element widely used in manipulating magnetic properties of molecules [1-5], hence, we developed [{(talen^t-Bu2^)Mn^III^_3_}_2_{Cr^III^(CN)_6_}]^3+ ^([**Mn^III^_6_Cr^III^]^3+^**) with H_6_talen^*t*-Bu2 ^= 2,4,6-tris(1-(2-(3,5-di-*tert*-butylsalicylaldimino)-2-methylpropylimino)-ethyl)-1,3,5-trihydroxybenzene [6-9]. This molecule was constructed using a supramolecular approach from three building blocks. Two identical bowl shaped trinuclear Mn^III ^complexes were bridged by a hexacyanochromate (Figure 1).

The strongest interaction is the antiferromagnetic coupling of the central Cr^III ^ion with the six terminal Mn^III ^ions which results in a spin ground state of the molecule of *S*_t _= 21/2. This high-spin ground state in combination with a strong easy-axis magnetic anisotropy and a *C*_3 _symmetry results in an energy barrier for spin-reversal, which leads to a slow relaxation of the magnetization at low temperatures (single-molecule magnetism behavior, i.e., molecular superparamagnetism [10,11]). [**Mn^III^_6_Cr^III^]^3+ ^**has a blocking temperature around 2 K [6,7]. Recent experimental spin resolved photoemission results of [**Mn^III^_6_Cr^III^]^3+ ^**single-molecule magnet (SMM) [12], X-ray magnetic circular dichroism (XMCD) at a Fe-SMM-adsorbed molecule [13] and cross-comparison between spin-resolved photoemission and XMCD in Mn-based molecular adsorbates have been published elsewhere [12]. The three positive charges of [**Mn^III^_6_Cr^III^]^3+ ^**can be neutralized by various anionic counterions. Herein, the three salts [**Mn^III^_6_Cr^III^**](BPh_4_)_3_, [**Mn^III^_6_Cr^III^**](C_3_H_5_O_3_)_3_, and [**Mn^III^_6_Cr^III^**](ClO_4_)_3 _were investigated using as three anions either tetraphenylborate (BPh_4_^-^), lactate (C_3_H_5_O_3_^-^), or perchlorate (ClO_4_^-^), respectively. Being able to choose between three different anions for the same core compound allowed us to study the influence of the anions with respect to the whole molecule-substrate-system.

Investigation in this regime is best done via non-contact atomic force microscope (AFM) [14,15]. Due to [**Mn^III^_6_Cr^III^]^3+ ^**simply physisorbing onto the surface, the use of non-contact (nc)-AFM allows us to observe the molecule with a decreased risk of manipulating the molecule during this process. Of special interest are the thin layers of [**Mn^III^_6_Cr^III^]^3+ ^**and whether these layers are crystalline or amorphous [16-19].

## Experiment

Preparation was carried out in air at room temperature (21 ± 1°C) and air moisture between 40% and 60% via the droplet technique using an amount of 10 μl and a concentration of 10^-5 ^mol/l of the solution. As the solvent, we used dichloromethane for [**Mn^III^_6_Cr^III^**](BPh_4_)_3 _and methanol for [**Mn^III^_6_Cr^III^**](C_3_H_5_O_3_)_3 _and [**Mn^III^_6_Cr^III^**](ClO_4_)_3_. Either the selected concentration and amount of solution, or the number of molecules, was sufficient for the creation of approximately one monolayer. During preparation the sample was held at an angle of 57° which led to a more homogeneous wetting. Substrates (10 × 10 mm^2^) were affixed onto Omicron carriers (Omicron NanoTechnology GmbH, Taunusstein, Germany).

The surface topography of the samples was analyzed by means of non-contact atomic force microscopy in ultra-high vacuum (UHV) (Omicron UHV-AFM/STM). The pressure of the vacuum chamber was approximately 10^-7 ^Pa and the measurements were taken at room temperature.

We used silicon non-contact cantilevers (NSC15, MikroMasch, San Jose, CA, USA) with a resonance frequency of approximately 325 kHz. The microscope was operated at a frequency shift between 20 and 80 Hz below the vacuum resonance frequency.

Image fields up to 720 × 720 nm^2^ were recorded with a scan speed of approximately 350 nm/s and 300 lines per image. Standard image processing was performed using a polynomial background correction by means of Gwyddion (version 2.19) and SPIP (version 5.0.6), in order to flatten the image plane.

The X-ray photoelectron spectroscopy measurements were recorded using a PHI 5600ci multitechnique spectrometer (Physical Electronics, Chanhassen, MN, USA) with a monochromatic Al K_α _(hν = 1,486.6 eV) radiation of 0.3 eV FWHM bandwidth. The sample was kept at room temperature. The resolution of the analyzer depended on the pass energy. During these measurements, the pass energy was 187.85 eV, leading to a resolution 0.44 eV. All spectra were obtained using a 400 μm diameter analysis area. During the measurements, the pressure in the main chamber was kept within the range of 10^-7 ^Pa.

The samples were oriented at a surface-normal angle of 45° to the X-ray source and -45° to the analyzer for all core-level X-ray photoelectron spectroscopy (XPS) measurements.

## Results

### HOPG

**Figure 2:**
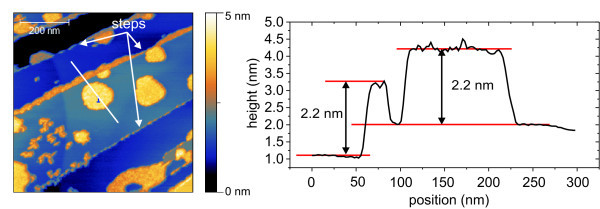
**Nc-AFM micrograph and image of [Mn^III^_6_Cr^III^](BPh_4_)_3_**. (**a**) Nc-AFM micrograph of [**Mn^III^_6_Cr^III^**](BPh_4_)_3 _on HOPG. 720 × 720 nm^2^ scan. 30% of the surface is covered with flat islands which are near the edges of the atomic steps. (**b**) Line scan of the nc-AFM image.

[**Mn^III^_6_Cr^III^**](BPh_4_)_3 _prepared on highly oriented pyrolytic graphite (HOPG) leads to flat island-like structures with height of about 2 nm. These structures appear in sizes from 10 nm diameter up to several hundred nanometers and even ones covering nearly the whole scanned area. Two main structures can be distinguished:

The first and more common way structures appear is shown in Figure 2. The islands cover approximately 30% of the surface and are mostly attached to an atomic step of HOPG. At the atomic step, an agglomeration of [**Mn^III^_6_Cr^III^**](BPh_4_)_3 _with average height of 2.2 nm occurs. The island shows also a height of 2.2 nm. It is not clear whether this is due to one layer of the stacking or two layers of [**Mn^III^_6_Cr^III^**](BPh_4_)_3_. The coverages can be divided into three groups:

1. Free islands which do not have any lateral contact. These show most often the tendency to appear in a circular manner.

2. Islands attached to a step edge. Again these tend to form a circle-like structure but are hindered by the edge. The islands do not continue their extension on the other side of the edge but seem to be cut off. No tendency can be seen as to whether these cut islands appear more often on the upper or lower side of the step edges.

3. Agglomeration along the step edges with no preference relating to upper or lower step edges.

**Figure 3:**
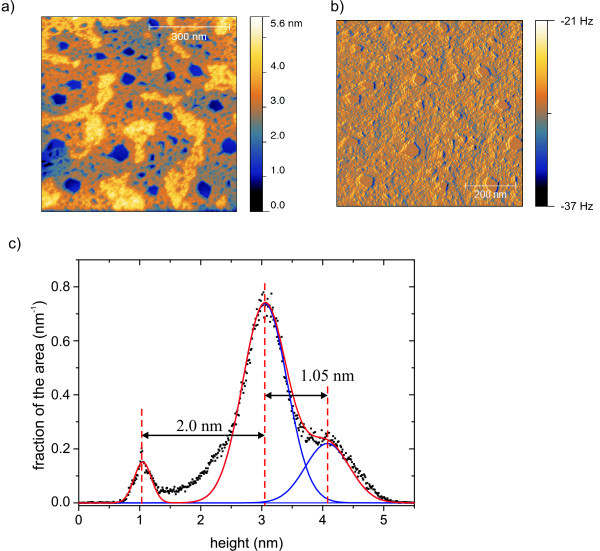
**Nc-AFM-images of [Mn^III^_6_Cr^III^](BPh_4_)_3 _on HOPG**. (**a**) Topography, (**b**) frequency shift. 95% of the area is covered by the molecules. 23% of the area is covered with a second layer. (**c**) Histogram of distribution of heights. Two plateaus are visible.

The second way [**Mn^III^_6_Cr^III^**](BPh_4_)_3 _appears is shown in Figure 3, where 95% of the whole area is covered with molecules. Two layers can be seen. The upper layer covers 23% of the surface. The layer thicknesses were estimated out of the histogram of the heights by Gaussian fits. The lower layer shows a height of 2.1 nm (see Figure 3c) while the upper layer is about 1.1 nm high and shows a higher rms roughness. Although the coverage of the area is nearly complete and even a second layer emerges on top of the first one, holes with diameters from 20 to 50 nm can be seen in the film. Because of a decreased roughness in these holes which become visible by the frequency shift image (Figure 3), we expect to see the plain substrate within the holes.

### Mica

**Figure 4:**
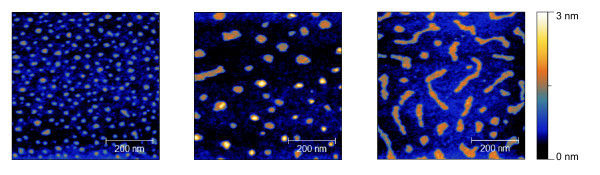
**Three nc-AFM-micrographs of [Mn^III^_6_Cr^III^](BPh_4_)_3 _on mica**. A gradient in the island size is visible.

On mica with [**Mn^III^_6_Cr^III^**](BPh_4_)_3_, a stronger influence of the preparation is visible due to a structural gradient. The gradient runs horizontally over the surface. We do not know whether there is also a vertical gradient, because of the limitations of the experimental setup. We divided this gradient into three stages:

1. In Figure 4 (left hand side), 9.8% of the area was covered by 316 [**Mn^III^_6_Cr^III^**](BPh_4_)_3 _particles. The average size of the particles was 11.9 nm at 161 nm^2^.

2. In Figure 4 (center), we moved along the gradient where the number of particles dropped down to 68, covering 8.4% of the surface. The mean particle size increased by a factor of 2 to 23.4 nm while the area covered rose to 640 nm^2^, and the particle height reached 1.1 nm.

3. In Figure 4 (right hand side), [**Mn^III^_6_Cr^III^**](BPh_4_)_3 _can be seen to form larger structures. The number of particles did not change. The covered area rose up to 17.1% while the average particle size reached 30.3 nm at 1270 nm^2^. Again, the height of the particles reached 1.1 nm leading to the conclusion that the gradient influences the covered area only and not the thickness of the layers.

### Silicon (SiO_2_, Si_3_N_4_)

We observed no difference in the investigated silicon-based materials such as SiO_2_, Si_3_N_4_. Furthermore, we used different oxide layers of SiO_2 _with thicknesses of 200 and 500 nm without any significant change.

**Figure 5:**
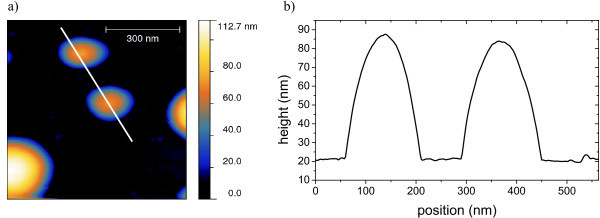
**[Mn^III^_6_Cr^III^](BPh_4_)_3 _prepared on Si_2_N_3 _in a concentration sufficient for one monolayer**. Occurrence of hemispheric clusters.

Large clusters appear with height from 10 to 100 nm. Even higher clusters may exist but these exceed the capabilities of the AFM in use. For clusters with height of about 55 nm, we observed diameters of up to 130 nm and clusters with a height of 80 nm showed a diameter of nearly 300 nm shown in Figure 5. In general the clusters appear to have a hemisphere-like form. In contrast to HOPG or mica, there are almost no small particles in between the bigger ones.

### Influence of the anions

Switching the anions to lactate on HOPG leads to a change in the emerging structures compared to the ones created with [**Mn^III^_6_Cr^III^**](BPh_4_)_3_. No islands are visible but the whole surface appears to be coated. It was not possible to measure the height of this film due to there being no trenches or other marks which would have allowed such an analysis. Due to non-existent islands, it is likely there is neither order in the film nor any kind of monolayer.

**Figure 6:**
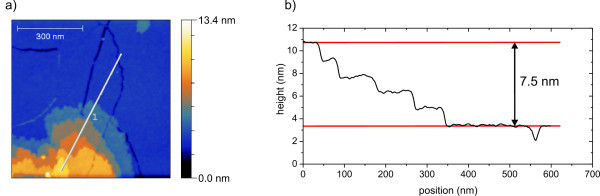
**Nc-AFM-micrograph of [Mn^III^_6_Cr^III^](C_3_H_5_O_3_)_3 _on mica**. Mica is fully covered by the molecules. (**b**) Line scan along the line displayed in (**a**). Five approximately equidistant steps can be observed in a range of 7.5 nm which is equivalent with a step height or a layer thickness of 1.5 nm.

**Figure 7:**
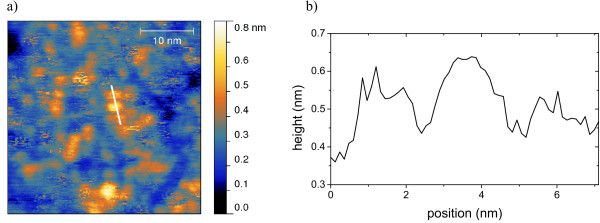
**Nc-AFM image of [Mn^III^_6_Cr^III^](C_3_H_5_O_3_)_3 _prepared on HOPG as a monolayer (**a**)**. (**b**) Line scan along the line displayed in (**a**).

The film-like structure also appears on mica as shown in Figure 6. The whole surface is coated with a layer of [**Mn^III^_6_Cr^III^**](C_3_H_5_O_3_)_3_. In this layer, trenches appear across the surface, which show depths of about 1.3 nm. This fits well with the height of the molecules. Nevertheless there are step-like clusters with up to five or more layers. Each of these layers shows height of about 1.5 nm, thus leading to the conclusion that these structures may originate from [**Mn^III^_6_Cr^III^**](C_3_H_5_O_3_)_3_, too. Figure 7a is a high-resolution nc-AFM micrograph of [**Mn^III^_6_Cr^III^**](C_3_H_5_O_3_)_3 _on HOPG, which shows circular structures in the magnitude of the [**Mn^III^_6_Cr^III^]^3+^** SMMs. From the line scan (Figure 7b), a distance of approximately 2.5 nm between the structures can be estimated.

**Figure 8:**
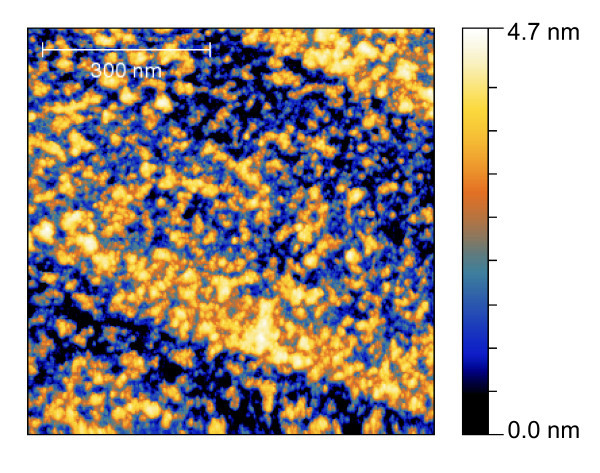
**Nc-AFM micrograph of [Mn^III^_6_Cr^III^](ClO_4_)_3 _on HOPG**.

Using (ClO_4_)_3_as the anion, the structures on HOPG appear like the ones seen using BPh_4 _but with fewer islands. These islands show height of about 1.4 nm. Nevertheless, parts of the sample are simply covered with randomly distributed deposited small particles (Figure 8). Most structures show a height of 1.1-1.4 nm.

**Figure 9:**
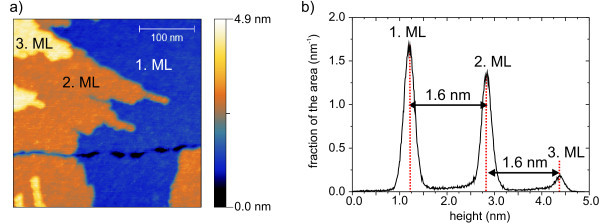
**Nc-AFM micrograph and image of [Mn^III^_6_Cr^III^](ClO_4_)_3 _on mica**. (**a**) Nc-AFM-micrograph of [**Mn^III^_6_Cr^III^**](ClO_4_)_3 _on mica. (**b**) Histogram of height of the nc-AFM image.

The structures evolving on mica look similar to the ones created by [**Mn^III^_6_Cr^III^**](C_3_H_5_O_3_)_3 _on mica. Multistep clusters with step sizes of 1.6 nm and trenches of 0.3 nm deep occur (Figure 9).

### XPS

We gained XPS spectra from [**Mn^III^_6_Cr^III^**](BPh_4_)_3 _and [**Mn^III^_6_Cr^III^**](ClO_4_)_3 _prepared as bulk and as monolayers. Data gained from the [**Mn^III^_6_Cr^III^**](BPh_4_)_3 _monolayer on HOPG is shown in Table 1. The values of the elements were normalized to the amount of six Mn atoms due to [**Mn^III^_6_Cr^III^]^3+ ^**containing six Mn atoms.

**Table 1:** XPS Data from **[Mn^III^_6_Cr^III^]**(BPh_4_)_3 _on HOPG

Element	Theoretical value	Measured value ± error
Cr	1	+0.56
		0.97
		-0.33
B	3	+1.2
		2.8
		-0.9
N	24	+6
		21
		-4
Normalized to Mn	6	6

XPS data of [**Mn^III^_6_Cr^III^**](BPh_4_)_3 _prepared as a monolayer on HOPG. Values are normalized to the amount of Mn.

## Discussion

### Influence of the substrate

The adsorption of any [**Mn^III^_6_Cr^III^]^3+ ^**salt is strongly influenced by the substrate on which it is prepared. Since [**Mn^III^_6_Cr^III^]^3+ ^**is a cation, it is crucial to neutralize its electric charge. In solution, the neutralization occurs through the anions which may move freely.

In the presence of a surface, we suggest the [**Mn^III^_6_Cr^III^]^3+ ^**trication could adsorb on the surface without the need of interaction with anions and bind to available adsorption sites on the substrate. An explanation for this speculation is the formation of mirror charges on the surface which assume the function of the anions.

We can divide the used substrate into two principal classes.

1. Molecule-substrate interaction being stronger than molecule-molecule interaction.

2. Molecule-substrate interaction being equal to or weaker than molecule-molecule interaction.

On the one hand, HOPG shows metallic properties which may allow [**Mn^III^_6_Cr^III^]^3+ ^**to build up mirror charges solely existing in the top graphene sheet causing a strong electrostatic interaction [20]. This would lead to the observed behavior of [**Mn^III^_6_Cr^III^]^3+ ^**trying to gain as much contact with the surface as possible. Nevertheless, this does not explain double-layers of [**Mn^III^_6_Cr^III^]^3+^**. As the trications would experience a strong electrostatic repulsion without interstitial anions, the close proximity of the anions in these double-layers appears to be very likely.

The interaction between the bottom [**Mn^III^_6_Cr^III^]^3+ ^**layer and the substrate may rely on the emerged mirror charges created by the positive charge of the SMM. This system is already stable at ambient conditions at room temperature. On HOPG we observe different heights for the first and second layer. This may be due to different van-der-Waals or mirror-charge interaction between two SMM layers in respect to the interaction between the substrate and the first SMM layer.

**Figure 10:**
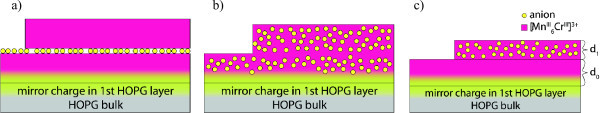
**Adsorption models of [Mn^III^_6_Cr^III^]^3+ ^on HOPG**. The first layer of the SMM is stabilized by mirror charges having their origin in the metallic HOPG substrate.(**a**) Model #1: Alternating stacking of SMM and anions. (**b**) Model #2: anions are in between the layer. (**c**) Model #3: similar to model #2 but the first layer is free of anions due to the mirror charge of the substrate thus leading to different heights of the first layer *d*_0 _and the consecutive ones *d*_1_...*d_n_*.

In the following, we present three models to show how [**Mn^III^_6_Cr^III^]^3+ ^**orders on top of HOPG (Figure 10).

### Model #1

#### SMM-Anion stacking

The first layer of the SMM is stabilized through the mirror charge. Thus a layer of anions can place itself on top of the [**Mn^III^_6_Cr^III^]^3+ ^**layer. By creating a negative charge at the surface, a second layer of [**Mn^III^_6_Cr^III^]^3+ ^**SMMs is attracted. If this is the case, it is unclear why this only takes place for a second layer of [**Mn^III^_6_Cr^III^]^3+^**. The anions can stabilize the SMM by themselves, thus the mirror charge created in the HOPG may simply be needed just at the start of the process. In this case, a second layer of anions is needed on top (Figure 10a).

### Model #2

#### Anions mixed with SMMs

It is more likely that a stronger interaction between the SMM and the anions leads to the anions being embedded inside a [**Mn^III^_6_Cr^III^]^3+ ^**layer. Also, this leads to lower levels of energy and higher levels of entropy inside the layer. However, we cannot distinguish whether the anions are needed in the bottom layer because of the mirror-charge effect. Nevertheless, we expect the anions to be in the top layer (Figure 10b).

### Model #3

#### Anions mixed with SMMs without anions in the first layer

Our results have shown a significant change in heights between the first and the following layers. This difference can be explained by a neutralization of charge of [**Mn^III^_6_Cr^III^]^3+ ^**caused by the mirror-charge effect in the first layer but by anions in the other ones (Figure 10c).

Mica on the other hand is an insulator, but being cleaved, the K^+ ^ions in the crystal are separated due to a weak binding to the close aluminosilicate [21] thus leading to surface potentials up to -130 V [22]. This potential becomes neutralized in air within a few minutes [22] but there are still enough negatively charged sites to allow [**Mn^III^_6_Cr^III^]^3+ ^**to adsorb at the surface. Further layers neutralize their charge the same way as with HOPG. Anions are in between the SMMs in one layer.

**Figure 11:**
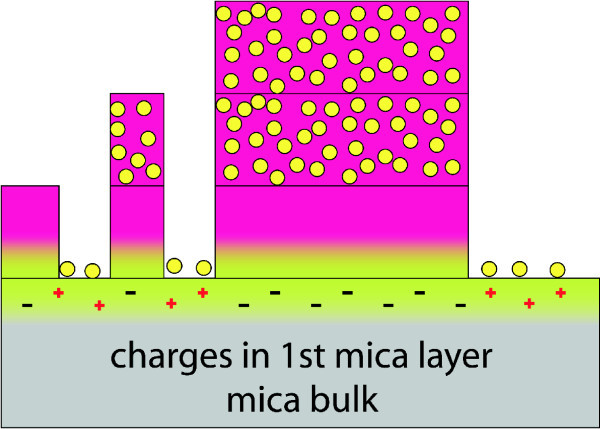
**Model of [Mn^III^_6_Cr^III^]^3+ ^including its anion on mica**. The positive SMM is attracted by negative charges localized at the surface of mica. Equally distributed positive charges attract the negatively charged anions. Due to this charge compensation there are no anions needed in the first layer. Consecutive layers require anions for charge neutrality which leads to the anions appearing inside these layers.

Two scenarios appear to be plausible which explain the observed gradient on mica. During the dropping of [**Mn^III^_6_Cr^III^**](BPh_4_)_3 _on top of the mica substrate, the tilted sample may have caused the gradient by an increased or decreased flow of the solution over the surface. The other explanation involves the surface charges of cleaved mica (Figure 11). It is known that these charges are distributed irregularly [22]. When being prepared using sticky film there is always one direction in which the film is ripped off. This may lead to a gradient in the K^+ ^ions left on the surface which influences the surface potential. [**Mn^III^_6_Cr^III^**](BPh_4_)_3 _follows the gradient of this distribution.

Using lactate or perchlorate as the anion, we have not yet been able to observe such a gradient. We expect the mobility of the anion to have an influence on the way [**Mn^III^_6_Cr^III^]^3+ ^**orders itself on the surface.

**Figure 12:**
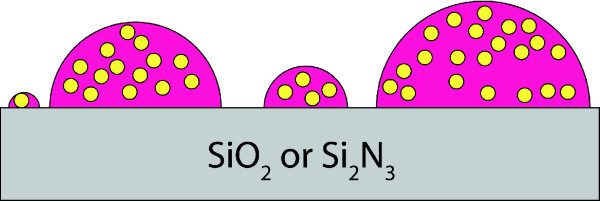
**Model of [Mn^III^_6_Cr^III^]^3+ ^including its anion on a Si-based substrate**. The substrate is an insulator and does not offer, unlike mica, any charges at the surface. This leads to the SMM and its anions minimizing the contact with the surface, which results in hemispheric-shaped clusters.

The second kind of substrate does not allow neutralization of charge except the one performed by the anions. This results in [**Mn^III^_6_Cr^III^]^3+ ^**minimizing the contact with the surface. The anions would try to minimize the contact with the surface for the same reason (Figure 12). Thus the increased surface energy leads to [**Mn^III^_6_Cr^III^]^3+ ^**and the respective anion used sticking together. The stoichiometry of the overall [**Mn^III^_6_Cr^III^]^3+ ^**salt including the anions may make it unattractive to place itself alone at the surface. In this respect, the most stable way of ordering appears to be in clusters. This explains why there is such a low influence on different silicon based substrates.

### Influence of the anions

The anions are crucial for the stability of the whole complex. As we have shown, changes in the anions may cause a drastic variation in the way [**Mn^III^_6_Cr^III^]^3+ ^**is absorbed on top of the surface.

The biggest difference can be seen between tetraphenylborate/perchlorate and lactate. The former ones show a strong influence by the substrate. Depending on which substrate is used various kinds of structures can be observed: flat islands, multistackings, big clusters, and even the homogeneous coverage of large areas. The latter shows just one structure. This is the coverage of the whole sample with an inhomogeneous but continuous film.

FFT performed on any of the systems did not reveal a crystalline structure resulting in [**Mn^III^_6_Cr^III^]^3+ ^**or its anions which is why we expect no epitactical growth.

### XPS

XPS data gained on [**Mn^III^_6_Cr^III^**](BPh_4_)_3 _confirmed the existence of a layer of the SMM on the HOPG surface.

The ratios between the elements, including four solvent molecules are close to the expected values for [**Mn^III^_6_Cr^III^**](BPh_4_)_3_. The errors of the ratios given in Table 1 are mainly due to the uncertainty of background substraction.

## Summary

We have demonstrated a strong influence of the electric properties of the used substrates on the ordering of [**Mn^III^_6_Cr^III^]^3+ ^**on the surface. Substrates allowing [**Mn^III^_6_Cr^III^]^3+ ^**to neutralize its charge cause more flat structures than the others on which [**Mn^III^_6_Cr^III^]^3+ ^**tends to form high clusters. Furthermore, we have investigated different anions used with [**Mn^III^_6_Cr^III^]^3+ ^**and observed a drastic change in occurrences on surfaces when lactate instead of tetraphenylborate or perchlorate is used.

## Competing interests

The authors declare that they have no competing interests.

## Authors' contributions

AG and HP carried out the AFM measurements supervised by AB and UH. CD carried out the XPS measurements supervised by MN. VH and EK synthesized the SMMs supervised by TG. All authors read and approved the final manuscript.
